# Peptide Aptamer PA3 Attenuates the Viability of *Aeromonas veronii* by Hindering of Small Protein B-Outer Membrane Protein A Signal Pathway

**DOI:** 10.3389/fmicb.2022.900234

**Published:** 2022-05-19

**Authors:** Peng Liu, Huimin Chang, Qi Xu, Dan Wang, Yanqiong Tang, Xinwen Hu, Min Lin, Zhu Liu

**Affiliations:** ^1^School of Life Sciences, Hainan University, Haikou, China; ^2^Center for Medical Innovation, School of Basic Medical Science, Guangxi University of Chinese Medicine, Nanning, China; ^3^One Health Institute, Hainan University, Haikou, China; ^4^Biotechnology Research Institute, Chinese Academy of Agricultural Sciences, Beijing, China

**Keywords:** *Aeromonas veronii*, SmpB, OmpA, peptide aptamer, antimicrobial-agent

## Abstract

The small protein B (SmpB), previously acting as a ribosome rescue factor for translation quality control, is required for cell viability in bacteria. Here, our study reveals that SmpB possesses new function which regulates the expression of outer membrane protein A (*ompA*) gene as a transcription factor in *Aeromonas veronii*. The deletion of SmpB caused the lower transcription expression of *ompA* by Quantitative Real-Time PCR (qPCR). Electrophoretic mobility shift assay (EMSA) and DNase I Footprinting verified that the SmpB bound at the regions of −46 to −28 bp, −18 to +4 bp, +21 to +31 bp, and +48 to +59 bp of the predicted *ompA* promoter (P*ompA*). The key sites C_52_AT was further identified to interact with SmpB when P*ompA* was fused with enhanced green fluorescent protein (EGFP) and co-transformed with SmpB expression vector for the fluorescence detection, and the result was further confirmed in microscale thermophoresis (MST) assays. Besides, the amino acid sites G11S, F26I, and K152 in SmpB were the key sites for binding to P*ompA*. In order to further develop peptide antimicrobial agents, the peptide aptamer PA3 was screened from the peptide aptamer (PA) library by bacterial two-hybrid method. The drug sensitivity test showed that PA3 effectively inhibited the growth of *A. veronii*. In summary, these results demonstrated that OmpA was a good drug target for *A. veronii*, which was regulated by the SmpB protein and the selected peptide aptamer PA3 interacted with OmpA protein to disable SmpB-OmpA signal pathway and inhibited *A. veronii*, suggesting that it could be used as an antimicrobial agent for the prevention and treatment of pathogens.

## Introduction

*Aeromonas veronii* is a serious pathogen which is dispersed in different geographical locations. It causes not only human diseases such as meningitis, diarrhea, and trauma infection (Cui et al., 2007; Prediger et al., 2020; Zhang et al., 2020), but also aquaculture diseases such as rot skin disease, septicemia, and rotten tail disease (Dong et al., 2017; Lazado and Zilberg, 2018; Liu Z. G. et al., 2018; Ran et al., 2018). In addition, *A. veronii* is resistant to many antibiotics together with penicillin, ampicillin, oxacillin and piperacillin (Yang et al., 2022). So it’s intractable to prevent and treat, and it is urgent to study the regulation mechanism of its virulence.

Ribosome rescue systems are pivotal to recycle arrested ribosomes that stall at the 3′ end of mRNAs, subsequently releasing the tagged polypeptide for degradation by cellular proteases and eventually permitting disassembly and liberating ribosomal subunits for new rounds of protein synthesis (Himeno et al., 2015). It is widely believed that *trans*-translation is the most ubiquitous and effective ribosome rescue system in bacteria (Konno et al., 2007), which is required for cell viability, growth, and pathogenesis (Brunel and Charpentier, 2016), and its inactivation decreases the abilities to respond to and recover from stress conditions (Yang and Glover, 2009), indicating that it may be a promising target for the development of new antimicrobial agents. The molecule inhibitor KKL-10 or KKL-40 exhibited antimicrobial activity against *Francisella Tularensis* through intervening *trans*-translation (Goralski et al., 2016). The small-molecule inhibitor of *trans*-translation KKL-40 also interacts with the human antimicrobial peptide LL-37 to inhibit *Staphylococcus aureus* (Huang et al., 2019). Small protein B (SmpB) is conserved and found as the binding partner of tmRNA (Hong et al., 2007). However, the SmpB and tmRNA have been shown to play distinct roles. The SmpB mutant exhibits higher susceptibility to antibiotics and stresses than the *ssrA* (the gene encoding tmRNA) mutant in *Escherichia coli* (Li et al., 2013). According to the reports, the *ssrA* regulates *S. aureus* pigment synthesis through base pairing with crtMN mRNA as an antisense RNA (Liu et al., 2010). So, does SmpB have other functions as well?

Most researchers believe that the SmpB act through *trans*-translation in virulence, but our previous study demonstrated that the SmpB interacted with the promoter of *bvgS* gene and enhanced the transcriptional expression without the participation of tmRNA in *A. veronii* (Liu et al., 2015). Here, we detailed again that the SmpB increased the expression of downstream outer membrane protein A (OmpA) as a transcription factor (TF), an effect that was entirely distinct from the previous *trans*-translation mechanism. OmpA protein is a highly conserved outer-membrane protein in Gram-negative bacteria. In addition to its role as a structural protein for the maintenance of cell integrity and morphology (Krishnan and Prasadarao, 2012), OmpA serves as a receptor for adhesion and invasion associated with virulence (Smith et al., 2007). Peptide aptamers (PAs), a kind of small combinatorial proteins, bind to specific sites on the target molecules. Compared with antibodies, PAs have advantages such as small size, simple and stable structure, and soluble expression in cells (Reverdatto et al., 2015). Owing to these advantages, PAs today are widely applied in bioimaging, targeted drug delivery, and the drug selection of antitumor and antimicrobial agents (Blum et al., 2000; Wang and Farokhzad, 2014; Zhou and Rossi, 2014; Tan et al., 2021). To evaluate the validation of the SmpB-OmpA signal pathway, the particular PA was selected utilizing OmpA as a bait in the bacterial two-hybrid system (Liu et al., 2016), followed by the interaction with OmpA and the growth inhibition. Collectively, we uncovered that SmpB-activated OmpA expression as a TF. The interactions identified in our study deepened the insight into SmpB-mediated regulation, unveiled the outer membrane biogenesis and identified the drug targets for the development of new inhibitors in bacteria.

## Materials and Methods

### Bacterial Strains and Plasmids

The strains, plasmids, and primers were defined in Supplementary Tables 1–3, respectively. The plasmids were constructed according to the conventional molecular biology techniques, such as PCR, DNA restriction, ligation, transformation, and positive colony selection (Aviv and Gal-Mor, 2018). Endonucleases and DNA polymerase were purchased from New England Biolabs (Ipswich, MA, United States). The DNA purification kit, oligonucleotide synthesis, and DNA sequencing were provided by Sangon Biotech (Shanghai, China). To construct the fluorescent reporter plasmid pDH116, both the promoter of the *ompA* gene and encoding region of enhanced green fluorescent protein (EGFP) were amplified by primers in Supplementary Table 3. The two DNA fragments were purified and combined to a fusion by overlap extension PCR and were subsequently inserted into the backbone of plasmid pDH113 (Liu et al., 2015).

### Media and Growth Conditions

Bacteria were cultured in LB broth or on LB agar plates at 37°C. A final concentration of 0.1 mM IPTG (isopropyl β-D-1-thiogalactopyranoside) was applied to induce the Lac or T7 promoter. Antibiotics were used at the following concentrations: 25 μg/ml of chloramphenicol, 100 μg/ml of ampicillin, 25 μg/ml of kanamycin, and 20 μg/ml of tetracycline. The culture was incubated at 37°C overnight with agitation and then inoculated in fresh medium until the exponential or stationary phase.

### Promoter Prediction

The upstream region from open reading frames (ORF) was uploaded and predicted by iPromoter-2L,^1^ BPROM,^2^ and Promoter Calculator.^3^ With multi-window-based PseKNC, iPromoter-2L is a two-layer predictor for identifying the sigma promoters in *E. coli*, such as σ^24^-promoter, σ^28^, σ^32^, σ^38^, σ^70^, and so on (Chen et al., 2014, 2015; Liu B. et al., 2018). BPROM is a bacterial sigma 70 promoter recognition program for annotating the upstream regions of ORFs (Solovyev and Salamov, 2011). Promoter Calculator is applied to predict promoter elements and transcriptional initiation rates across transcriptional start sites (TSSs) depending on free energy calculation.

### Growth Measurement and Fluorescent Analysis

Bacterial growth was monitored by taking 1 ml of culture to measure the optical density (OD) at 600 nm using a spectrophotometer (Shanghai Spectrum Instruments Co., Ltd., Shanghai, China) at appropriate times. The total fluorescence was determined by analyzing 200-μl aliquots of culture with a fluorescent plate reader by Infinite^®^ 200 PRO instrument (Tecan, Shanghai, China), wherein the wavelengths of excitation and emission were programmed at 485 and 525 nm, respectively. Each treatment was performed in triplicate with separated bacterial cultures, and the fluorescence values were normalized to the OD.

### Bacterial One-Hybrid and Two-Hybrid Assays

Bacterial one-hybrid assay was described previously (Guo et al., 2009). Briefly, the *E. coli* XL1-Blue MRF′ strain was applied to the propagation of all pBX-cmT and pTRG recombinant plasmids. A pair of pBX-cmT and pTRG derivatives was co-transformed into the *E. coli* XL1-Blue MRF′ reporter strain for the growth test. The principle of DNA–protein interactions was dependent on the transcriptional activation of the *HIS3* reporter gene, which allowed the growth in the presence of 3-amino-1,2,4-triazole (3-AT), a competitive inhibitor of His3 enzyme. Besides, the positives were verified by using the aadA gene, which conferred streptomycin resistance, as a secondary reporter. Collectively, the selective medium was appended with 3-AT, streptomycin, tetracycline (resistance of pTRG and its derivative plasmids), chloramphenicol (resistance of pBT and its derivative plasmids), and kanamycin (resistance of *E. coli* reporter strains) (Guo et al., 2009). Likewise, two-hybrid assay was performed except that the conjugated plasmids were pBT and pTRG derivatives (Strauch and Georgiou, 2007).

### Quantitative Real-Time PCR Assays

The strains were cultured in M9 minimal medium (standard). Briefly, total RNA was extracted from all strains, and the residual DNA was removed by DNase I treatment (Green and Sambrook, 2018). First-strand complementary DNA (cDNA) was synthesized employing the HiScript^®^ IIQ RT SuperMix (Vazyme, Nanjing, China) and was subsequently diluted as the template for the quantitative real-time PCR (qPCR) analysis. The primers used in qPCR were listed in Supplementary Table 4. The qPCR reaction was conducted using the LightCycler^®^ 96 instrument (Roche, Switzerland, Germany) for the fluorescence detection. The relative amounts of RNAs were calculated using the comparative Ct method by selecting Glyceraldehyde-3-phosphate dehydrogenase (GAPDH) as the reference gene (Schmittgen and Livak, 2008).

### Electrophoretic Mobility Shift Assays

To prepare the DNA probe, a 125-bp fragment of the predicted *ompA* promoter (P*ompA)* region was amplified from the pDH116 plasmid with primers that had been labeled by 6-carboxyfluorescein (6-FAM) (Xu et al., 2017). To prepare the SmpB protein, the pET-28a-SmpB was transferred into *E. coli* BL21. The SmpB was purified as the calculated molecular weight of 19 kDa using NI-IDA column. For each assay, 40 ng of probe was incubated with increasing amounts of SmpB protein in a total volume of 20 μl. After incubation at 30°C for 30 min, the samples were loaded onto 5% polyacrylamide native gels for electrophoresis in 0.5 × TBE running buffer at 10 V/cm for approximately 1.5 h. The DNA fragment bound to SmpB protein was detected using the Image Quant LAS 4000 mini system (GE Healthcare, Piscataway, United States).

### DNase I Footprinting Assay

After the DNA probe and SmpB protein were mixed and incubated as previously described EMSA, 5 μl of solution including 0.015 units of DNase I (Promega, Madison, United States) and 100 nmol of CaCl_2_ were added, followed by further incubation for 1 min at 25°C. The reaction was terminated by adding 70 μl of DNase I stop solution. The samples were extracted with phenol/chloroform, precipitated with ethanol, and dissolved in 20 μl of Mini-Q water. To prepare the DNA ladders, the fmol DNA cycle sequencing system (Promega, Madison, United States) was applied. Twelve microliters of the sequencing reactions were performed using 15 ng of the P*ompA* region as the template and 5 pmol of FAM-labeled oligonucleotides as the sequencing primer. Subsequently, 1 μl of digested DNA fragments was added to 8.5 μl of highly deionized (HiDi) formamide and 0.5 μl of GeneScan-LIZ600 size standards (Applied Biosystems, Foster City, United States), and then the mixture was analyzed using the 3130xl DNA analyzer and Peak Scanner software v1.0 (Applied Biosystems, Foster City, United States) (Cao et al., 2017).

### Microscale Thermophoresis

The purified SmpB protein was labeled by the RED-tris-NTA 2nd Generation dye (NanoTemper Technologies, Munich, Germany) to obtain the final concentration of 50 nM fluorescent molecules. The DNA fragments including the native P*ompA* (50 μM P*ompA* C_52_AT) and the mutation (800 μM P*ompA* T_52_GC) were used as the ligand molecules. The microscale thermophoresis (MST) reaction buffer was phosphate buffered saline-tween (PBS-T) buffer (2 mM KH_2_PO_4_, 8 mM Na_2_HPO_4_, 350 mM NaCl, 0.6 mM DTT, 0.05% Tween 20, and pH 7.4). Approximately, 10 μl of the serial diluted ligand molecules was mixed with the equal volume of fluorescent molecules. The reaction products were sampled in a standard capillary and placed in a MO NT.115 capillary tray (NanoTemper Technologies, Munich, Germany). The samples were measured at 60% LED/excitation power and medium MST power. The *K*_*D*_ value was calculated by the MO.Affinity Analysis software (NanoTemper Technologies, Munich, Germany).

### Selection of Peptide Aptamers That Tnteracts With Outer Membrane Protein A

The peptide aptamer library (pTRG-SN-peptides) was constructed, and each PA comprised a scaffold protein *S. aureus* nuclease (SN) and a random peptide which exposed as a surface loop and bulged out on the surface of the scaffold. The plasmid pBT-*ompA* was applied as a bait to screen specific PA interacting with OmpA by the bacterial two-hybrid assay. The interactive key regions between OmpA and PA were identified as described previously (Liu et al., 2016).

### Susceptibility Test of the Peptide Aptamers in *Aeromonas veronii*

The peptide aptamer PA3 with molecular weight of 21 kDa that interacted with OmpA was expressed and purified for further use in *E. coli.* The susceptibility test was performed using the broth dilution method. *A. veronii* was grown in LB broth overnight at 30°C, and 3 ml of culture was diluted until an OD_600_ of 0.05, in which the purified peptide aptamer PA3 was supplemented at a final concentration of 50 μg/ml. After incubation for 10 h at 30°C, the OD_600_ of *A. veronii* was measured. In the meantime, phosphate buffered saline (PBS) buffer and bovine serum albumin (BSA) and purified SN were chosen as the negative controls, and chloramphenicol (Cm) was chosen as the positive control. The purified peptide aptamer PA3 was serially diluted at a range of 3–50 μg/ml.

### Statistical Analysis

The result data were based on at least three independent experiments and were displayed as the means ± standard deviation. The statistical significance was determined using one-way ANOVA, where * and ^**^ represented significant (*p* < 0.05) and extremely significant differences (*p* < 0.01), respectively.

## Results

### Small Protein B Binds to the *ompA* Promoter *in vitro*

**FIGURE 1:**
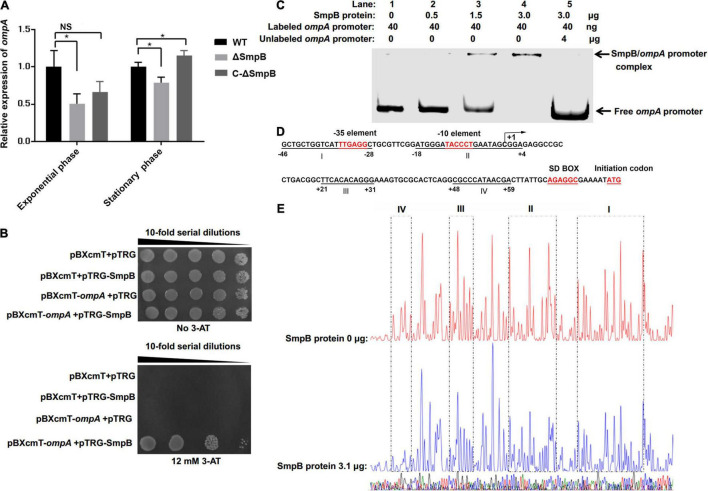
Identification of the key region in the predicted *ompA* promoter (P*ompA*) interacting with small protein B (SmpB). **(A)** Quantitative real-time PCR (qPCR) assays. The relative expression of *ompA* was analyzed in WT, ΔSmpB, and C-ΔSmpB in the exponential and stationary phase, respectively. The NS represents “not statistically significant” and * represents significant differences (*p* < 0.01). **(B)** Verification of SmpB interacting with the P*ompA* by bacterial one-hybrid system. The interaction between SmpB and the P*ompA* was monitored by the growth on 3-AT-free and 12 mM 3-AT medium. **(C)** Binding of SmpB to the P*ompA*. Electrophoretic mobility shift assay (EMSA) was performed using the purified SmpB with the promoter regions of the *ompA* gene. The DNA segments were amplified from the promoter of the predicted *ompA* gene. **(D)** The DNA sequences of the protected regions were provided, and the sequences were underlined. Transcriptional start site was marked with an arrow. Nucleotide sequences were numbered considering the transcription initiation site as +1. **(E)** Identification of SmpB-protected DNA fragments in the promoter regions of *ompA* using DNase I footprinting assays. The regions protected by SmpB were marked with frames of dashed lines.

In the previous study, the interference of PA on the function of SmpB resulted in the down-regulation of *ompA* transcriptional level in *A. veronii* (Liu et al., 2017). To analyze whether SmpB regulated OmpA directly, qPCR was performed in WT (wild type), ΔSmpB (SmpB deletion strain), and C-ΔSmpB (complementing *smpB* to ΔSmpB) at exponential phase and stationary phase, respectively. The transcription levels of *ompA* in ΔSmpB strain were lower at both phases compared to WT, while those of C-ΔSmpB strain had no difference at exponential phase and had significant increase at stationary phase (Figure 1A). We speculated that there might be an unknown TF that assisted SmpB protein to regulate the expression of OmpA and played unlike roles in different developmental stages of *A. veronii*. Whether this TF directly acted on OmpA or indirectly affected OmpA by regulating the expression of SmpB protein needed further study. Bacterial one-hybrid assays were performed to analyze the interaction between SmpB and the P*ompA*. The results showed that the co-transformants conferring SmpB and the P*ompA* were grown in the presence of 12 mM 3-AT media (Figure 1B and Supplementary Figure 1), indicating that SmpB specifically bound to the P*ompA* region *in vitro*. However, the interaction of SmpB and OmpA could not be observed by the bacterial two-hybrid assay (Supplementary Figure 2).

In addition, gel shift assay was performed to verify the binding between SmpB and the promoter of *ompA*. The 6 × His-tagged SmpB in the range of 0–3 μg was initially incubated with 40 ng of the labeled P*ompA* (Figure 1C), in which DNA was completely bound with 3 μg of SmpB. When 4 μg of unlabeled probes was added to the reaction, the overloaded DNA was competitive to form the complex with SmpB, resulting in a surplus of labeled DNA and the disappearance of the labeled DNA–SmpB complex (Figure 1C). The upstream elements before *ompA* ORF of *ompA* gene were predicted by iPromoter-2L (Figure 1D), of which including a σ^70^ promoter sequence. In addition, −10 element, −35 element, and TSS were deduced by BPROM and Promoter Calculator (Supplementary Figure 3 and Supplementary Dataset 1). Since the average nucleotide number of paired mRNA: 16S rRNA was 6.3 in bacteria 33 (Schurr et al., 1993) and the sequence (AGGAGG) was confirmed as Shine–Dalgarno (SD) sequence for promoting translation (Vimberg et al., 2007), a similar SD sequence (AGAGGC) was exhibited at the P*ompA* region in *A. veronii*. Subsequently, the SmpB-protected DNA sequences were precisely determined by the DNase I footprinting assay (Figure 1E). Four protected regions were located at −46 to −28 bp, −18 to +4 bp, +21 to +31 bp, and +48 to +59 bp, respectively, which were located in the upstream of the initiation codon of *ompA* (Figure 1D).

### Small Protein B Positively Regulates Outer Membrane Protein A Expression at the Stationary Stage

**FIGURE 2:**
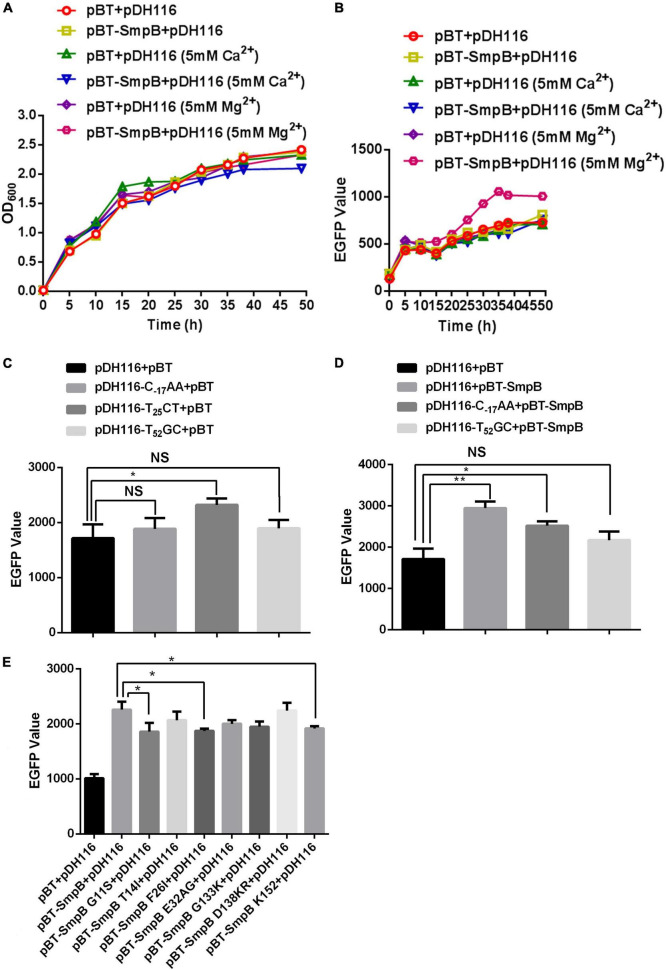
Small protein B regulates the transcription of P*ompA* positively. **(A)** Growth measurement of the strain bearing pDH116 when co-transformed with either pBT-SmpB or empty vector pBT. The plasmid pDH116 is endowed with both the promoter of the *ompA* gene and encoding region of enhanced green fluorescent protein (EGFP). **(B)** Fluorescence measurement of the strain bearing pDH116 when co-transformed with either pBT-SmpB or empty vector pBT. **(C)** Promoter activity of the native and mutated pDH116 (pDH116-C_–17_AA, pDH116-T_25_CT, and pDH116-T_52_GC). **(D)** Effects of SmpB on the expression of the native and mutated pDH116. **(E)** Identifications of the key amino acid residues in SmpB interacting with pDH116. The “NS” represents “not statistically significant” and “*,**” is significant (*p* < 0.05) and extremely significant differences (*p* < 0.01), respectively.

To further investigate the regulation of OmpA by SmpB, we monitored the fluorescence activities of the P*ompA* and EGFP translational fusion in the presence or absence of SmpB expression. It has been reported that divalent metal ion Mg^2+^ or Ca^2+^ stabilized the molecule conformation and affected interactions through neutralizing the negative charges (Lusetti et al., 2003; Onoda et al., 2005; Ralec et al., 2017; Kim et al., 2018). Hence, divalent metal ion Mg^2+^ or Ca^2+^ was added and the results demonstrated all the cells maintained constant growth (Figure 2A), while the cells expressing SmpB showed the significant fluorescent enhancement when supplemented with 5 mM Mg^2+^ compared with the controls (Figure 2B), signifying Mg^2+^ may help to stabilize the binding of protein and DNA structure. To examine the contribution of each binding site in the P*ompA* region, the point mutations were introduced to disrupt the binding of SmpB. Based on the experimental results of DNase I footprinting assay, the sites T_–17_GG, C_25_AC, and C_52_AT in the P*ompA* were mutated to C_–17_AA, T_25_CT, and T_52_GC, respectively. After the native and mutated reporters were transformed separately, the fluorescent levels were measured to analyze the promoter activities at the stationary stage. The results revealed that C_–17_AA and T_52_GC mutations themselves had no effect on the promoter activities, while the promoter activity was changed by T_25_CT mutation, which was abnegated afterward (Figure 2C). When SmpB and the promoter were co-transformed, the cells containing native promoter or C_–17_AA mutation increased the reporter expression, while T_52_GC mutation did not, indicating that the site was required for SmpB regulation (Figure 2D). Next, SmpB was truncated and mutated to analyze the effects of the P*ompA*. The results revealed that the G11S, F26I, and K152 sites of SmpB were vital to interact with the P*ompA* (Figure 2E).

### The C_52_AT Site of the *ompA* Promoter Interacts With Small Protein B by Microscale Thermophoresis Assays

**FIGURE 3:**
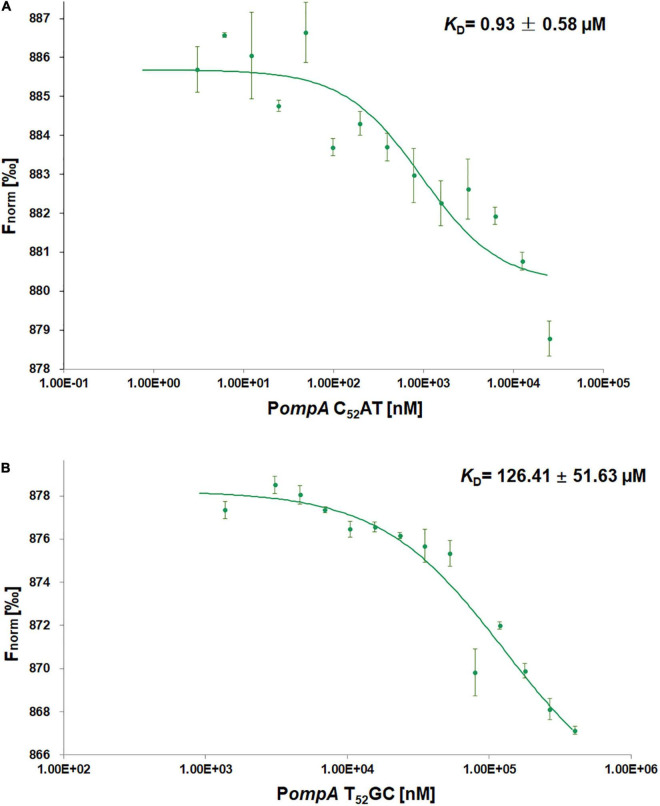
Small protein B protein interacts with the P*ompA* by microscale thermophoresis (MST) assays. **(A,B)** Dose Response Fit of SmpB protein with P*ompA* C_52_AT and P*ompA* T_52_GC. Fnorm values of Y-axis in the dose response plot are calculated from the ratio of normalized fluorescence F_0_/F_1_, where F_0_ corresponds to the normalized fluorescence prior to MST activation. F_1_ is determined after an optimal MST power-dependent time interval which yields the best signal-to-noise ratio. The X-axis is the different concentration of P*ompA* after successive dilution. The initial concentrations of P*ompA* C_52_AT (native *ompA* promoter) and P*ompA* T_52_GC (mutant) were, respectively, 25 and 400 μM in reaction buffer and the fluorescence molecules SmpB labeled by Monolith His-tag Labeling Kit RED-tris-NTA 2nd Generation was 25 nM in the buffer. MST experiments were performed in Phosphate buffered saline-Tween (PBS-T) buffer (2 mM KH_2_PO_4_, 8 mM Na_2_HPO_4_ 350 mM NaCl, 0.6 mM DTT, 0.05% Tween 20, pH 7.4) with 40% MST-Power, 60% Excitation-Power).

The initial concentrations of the native *ompA* promoter (50 μM P*ompA* C_52_AT) or the mutant (800 μM P*ompA* T_52_GC) were serially diluted, and mixed in equal volume with the labeled SmpB probes (50 nM) to obtained the dose–response fit graph. The *K*_*D*_ values were calculated using MO. Analysis Software. The *K*_*D*_ value of P*ompA* C_52_AT binding with SmpB was 0.93 ± 0.58 μM (Figure 3A), while that of the mutation P*ompA* T_52_GC binding with SmpB was 126.41 ± 51.63 μM (Figure 3B). The affinity of the P*ompA* with SmpB protein decreased significantly after C_52_AT was mutated to T_52_GC, demonstrating that C_52_AT site was the key site for SmpB binding to the P*ompA* region.

### Screen of Peptide Aptamers Interacting With Outer Membrane Protein A by the Bacterial Two-Hybrid System

**FIGURE 4:**
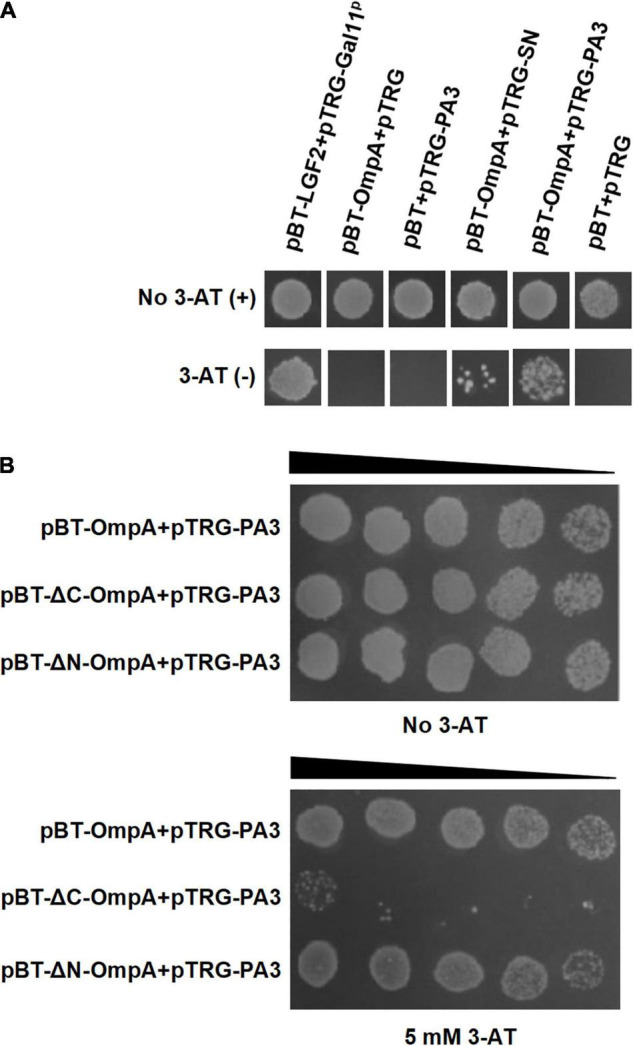
Interaction between OmpA and the specific peptide aptamer PA3. **(A)** The co-transformants were cultivated overnight and were spotted onto no 3-AT or 5-mM 3-AT media with 10 μl of 10^6^ CFU/ml. **(B)** Identification of the key regions of OmpA interacting with PA3 by the bacterial two-hybrid system.

The bacterial two-hybrid system was performed to identify PA that bound specifically to OmpA protein *in vivo*. Six clones that might interact with OmpA were isolated from 2 × 10^2^ transformants (Table 1). The colony designated as PA3 exhibited the strongest interaction with OmpA (Figure 4A). To further explore the key region of OmpA that interacted with PA3, OmpA truncations including pBT-ΔN-OmpA and pBT-ΔC-OmpA, were co-transformed with pTRG-PA3. The results showed that C-terminal OmpA bound to PA3 specifically (Figure 4B).

**TABLE 1:** Sequences of peptide aptamers.

Aptamer	Variable region
PA3	GGLRCWAVAFHARRNHCRGG
PA4	GGSCPTHLERNRSKYGTNGG
PA5	GGASQADMRGRVPVTEDHGG
PA6	GGPGVHGPLEQQREAGDEGW
PA7	GGLKRPALLAHADVGGLRGW
PA8	GGVTFLVNTYPNGVQSRAGG

### Antimicrobial Activity of the Peptide Aptamer PA3 *in vitro*

**FIGURE 5:**
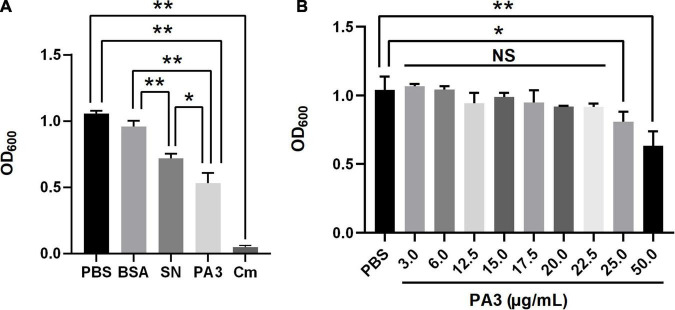
Characterization of peptide aptamer PA3. **(A)** Antibacterial activity of PA3 against *Aeromonas veronii.* Each of phosphate buffered saline (PBS), bovine serum albumin (BSA), SN, PA3, or Cm was added to the bacterial cultures, and OD_600_ measurements were carried out after 10 h of incubation. **(B)** Susceptibility test of PA3 in *A. veronii*. The serial concentrations of PA3 were diluted for supplementation in the bacterial cultures, and OD_600_ measurements were carried out after 10 h of incubation. The “NS” represents “not statistically significant” and “*,**” are significant (*p* < 0.05) and extremely significant differences (*p* < 0.01), respectively.

To confirm the antimicrobial activity of PA3, the growth assay was performed to test the inhibition of *A. veronii*. The results showed that the final concentration of purified PA3 was above 25 μg/ml; it could significantly inhibit the growth of *A. veronii* (Figure 5). These results demonstrated that PA3 could effectively inhibit the growth of *A. veronii*, suggesting PA3 possibly interfered the function of OmpA protein through the interaction with OmpA.

## Discussion

Outer membrane protein A maintains the structural integrity of the outer membrane together with cell wall lipoprotein and peptidoglycan in Gram-negative bacteria and contributes to nutrient absorption, pathogenicity, and multiple drug resistance (Vàzquez-Juárez et al., 2004; Confer and Ayalew, 2013). In addition, OmpA mediates the adhesion of pathogenic strains to leukocytes and macrophages, and the addition of exogenous OmpA antibody significantly reduces the adhesion to cells (Krishnan and Prasadarao, 2012). However, few studies are related to the regulation of OmpA.

Here, we reported a novel mechanism of SmpB participating in the regulation of outer membrane biogenesis of OmpA in *A. veronii*. SmpB is principally considered as a ribosome rescue factor, which works with tmRNA for quality control and dissociation of stalled ribosomes. The deficient SmpB attenuates growth, colonization, virulence, and antibiotic persistence (Mu et al., 2013; Liu et al., 2017; Mraheil et al., 2017). Apart from ribosome rescue, the action of SmpB alone was first shown to increase the *bvgS* expression as a TF without the participation of tmRNA (Liu et al., 2015). This work, building on our prior work, revealed that SmpB was required to bind to the promoter region of *ompA* (Figures 1, 2), which functioned in a manner similar to *bvgS* activation in *A. veronii* (Liu et al., 2015). The P*ompA* C_52_AT of *ompA* promoter and the G11S, F26I, and K152 of SmpB were the key sites for association. However, the protein interaction of SmpB and OmpA could not be observed by the bacterial two-hybrid assay (Supplementary Figure 2). These results suggested that SmpB-regulated *ompA* at the gene level. Whether SmpB alters the P*ompA* structure to make RNA polymerases or ribosomes more accessible still need to be further researched.

To clarify the importance of OmpA in *A. veronii*, a particular PA was identified to interact with OmpA for interfering with its function. PA exerts the intrinsic properties of antibody with small size, leading to convenient production and application (Blum et al., 2000; Gebauer and Skerra, 2009). PA3 screened from PA library exhibited strong interaction with OmpA. In the presence of 50 μg/ml of purified PA3, the growth of *Acinetobacter baumannii* was significantly inhibited compared with the control (Figure 5). The above results are similar to that the cyclic hexapeptide binding with OmpA inhibits its function and enhances the activity of colistin against *A. baumannii* (Parra-Millán et al., 2018). As the main component of Omp in Gram-negative bacteria and a key virulence factor of pathogenic bacteria, OmpA plays an important role in mediating bacterial biofilm formation, antibiotic resistance, and cell infection (Nie et al., 2020), which indicating that OmpA may serve as a potential drug target for combating *A. veronii* infection.

**FIGURE 6:**
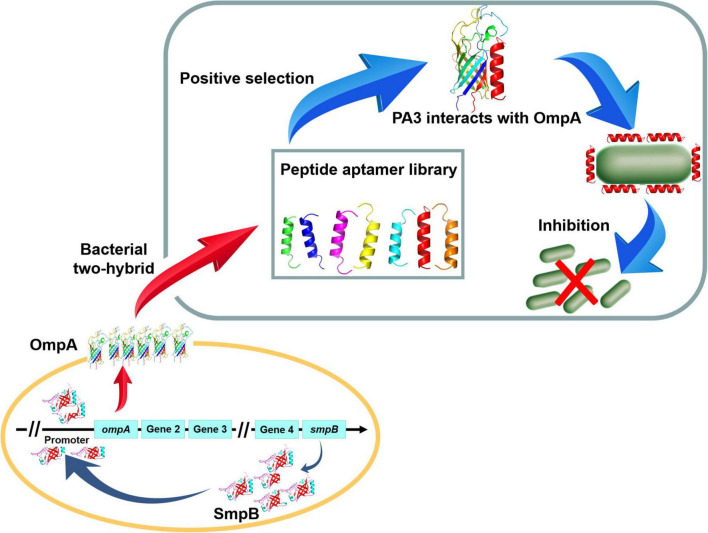
The schematic diagram of Peptide aptamer PA3 attenuates the viability of *A. veronii* by hindering of SmpB-OmpA signal pathway.

In summary, a possible mechanism for SmpB-mediated regulation of OmpA biogenesis in *A. veronii* was proposed based on multiple molecular biology techniques (Figure 6). Notably, the downstream node OmpA seemed terminal in the regulatory network because the inhibition of OmpA could hamper the pathway and seriously influence the growth by the PA (Table 1). We postulate that OmpA is a potential drug target for human and animal diseases.

## Data Availability Statement

The original contributions presented in the study are included in the article/Supplementary Material, further inquiries can be directed to the corresponding author.

## Author Contributions

PL and HC conceived the study, designed and performed the experiments, analyzed the data, and prepared the manuscript. QX, DW, and YT assisted in data analysis. XH and ML assisted in manuscript editing. ZL contributed to experimental design, data analysis, and preparation and editing of the manuscript. All authors have read and approved the manuscript.

## Conflict of Interest

The authors declare that the research was conducted in the absence of any commercial or financial relationships that could be construed as a potential conflict of interest.

## Publisher’s Note

All claims expressed in this article are solely those of the authors and do not necessarily represent those of their affiliated organizations, or those of the publisher, the editors and the reviewers. Any product that may be evaluated in this article, or claim that may be made by its manufacturer, is not guaranteed or endorsed by the publisher.

## Footnotes

1
http://bliulab.net/iPromoter-2L/

2
http://www.softberry.com/berry.phtml?topic=index&group=programs&subgroup=gfindb

3
https://salislab.net/software/predict_promoter_calculator
