# Supplementary material for: Peptide Aptamer PA3 Attenuates the Viability of Aeromonas veronii by Hindering of Small Protein B-Outer Membrane Protein A Signal Pathway
Source: Front Microbiol. 2022 May 19;13:900234. doi: 10.3389/fmicb.2022.900234 (PMC9159911; doi:10.3389/fmicb.2022.900234)
Supplement: Supplementary file 2 [file Data_Sheet_2.docx]

Supplementary Material

Supplementary Figure Legends


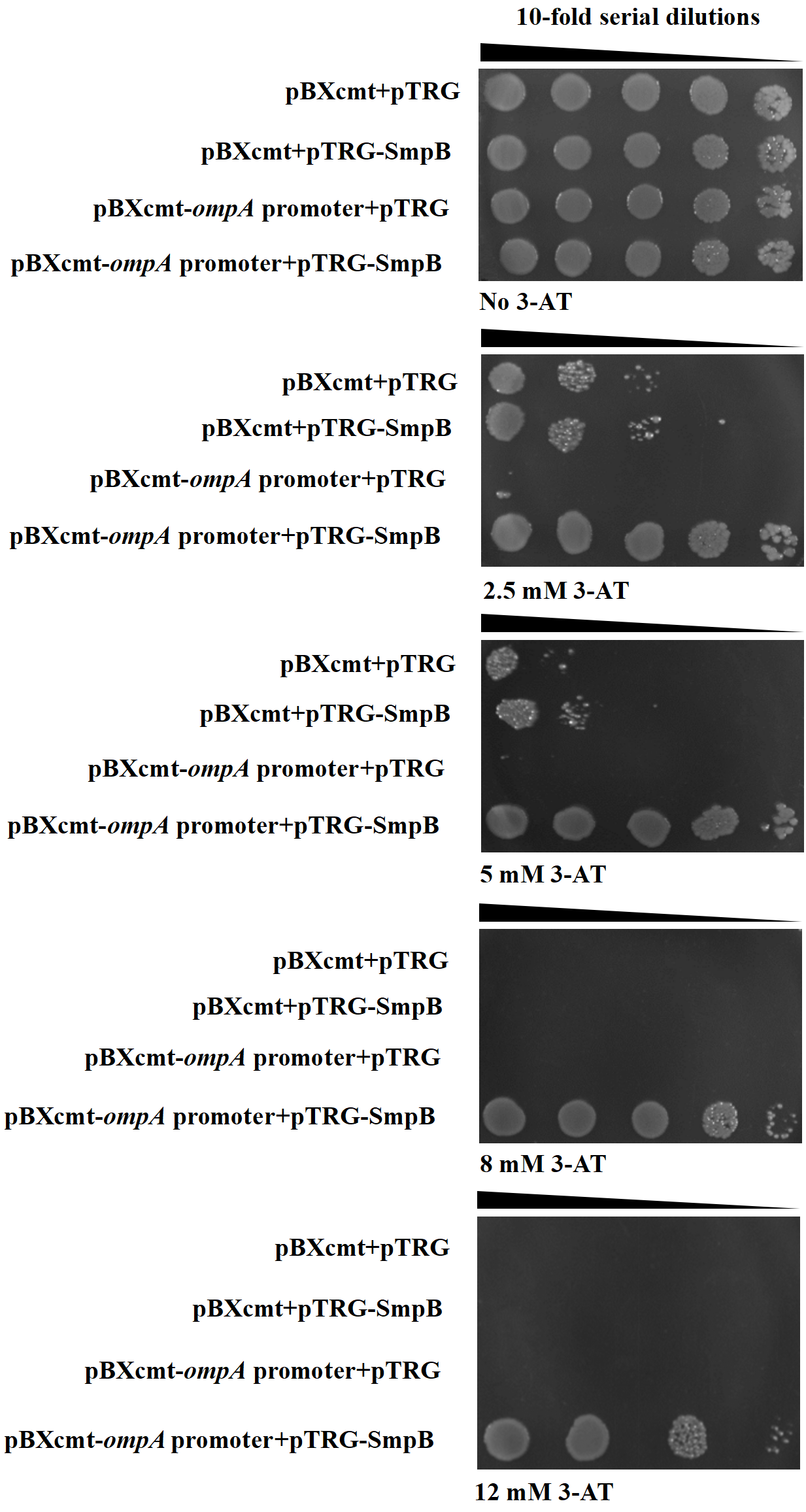


Supplementary Figure. S1. Identification of the interaction between *Aeromonas veronii* C4 SmpB protein and *ompA* promoter by bacterial one-hybrid system. The interaction between SmpB and *ompA* promoter was monitored by growth on NO 3-AT and 5-12 mM 3-AT.


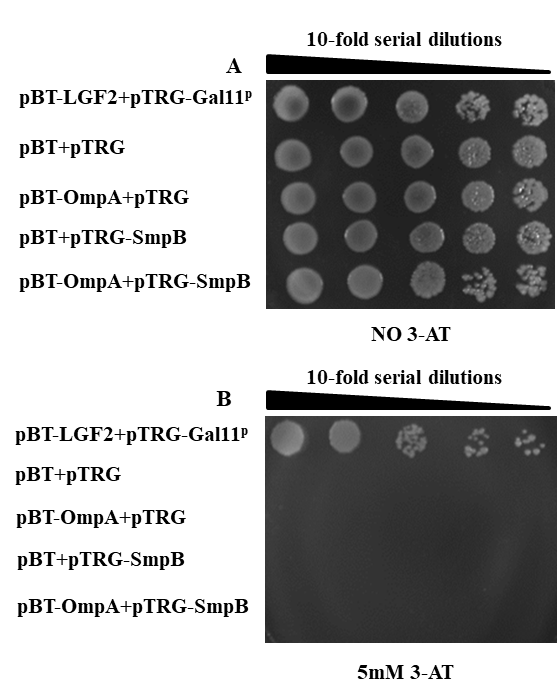


Supplementary Figure. S2. The bacterial two-hybrid system verified that there was no interaction between SmpB and OmpA. The interaction between SmpB and OmpA was monitored by growth on NO 3-AT (A) and 5 mM 3-AT (B).


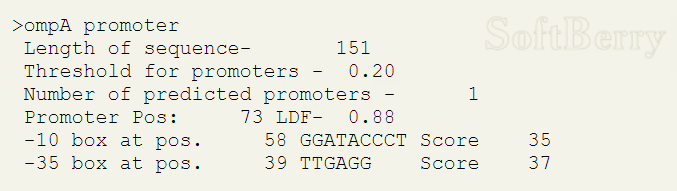


Supplementary Figure S3. Prediction of *ompA* gene promoter. The upstream region of the open reading frame (ORF) of *ompA* gene was input into BPROM, a -10 box and -35 box were detected, and the sequences were GGATACCCT and TTGAGG, respectively.

Supplementary Table Legends

Supplementary **Table S1** Bacterial strains used in this study

| **Strain** | **Description** | **Sources** |
| --- | --- | --- |
| *Aeromonas veronii* C4 | Wild type, ampicillin resistance, virulent to fish. | (1) |
| *A. veronii* Δ*smpB* | Ampicillin resistance, *smpB* gene was deleted from WT. | (1) |
| *A. veronii* C-Δ*smpB* | Ampicillin resistance, complementing *smpB* gene to Δ*smpB* | This study |
| *E. coli* XL1-Blue MRF' | Propagates pTRG, pBT , pBX-cmT and their derivatives, kanamycin resistance | Keysight Technologies Inc, Santa Clara, CA |
| *E. coli* XL1-Blue MRF' reporter strain | Expresses pTRG, pBT , pBX-cmT and their derivatives for two-hybrid and one-hybrid systems, kanamycin resistance | Keysight Technologies Inc, Santa Clara, CA |
| *E. coli* WM3064 | encodes a relaxase, a mating pair formation (MPF) complex and a type IV coupling protein in chromosome, diaminopimelic acid (Dap) auxotroph. | (2) |
| *E. coli* BL21 (DE3) | Protein expression host | New England Biolabs, Ipswich, MA |

Supplementary **Table S2** Plasmids used in this study

| **Plasmid** | **Description** | **Source** |
| --- | --- | --- |
| pRE112 | Suicide plasmid for gene knock out, chloramphenicol resistance, includes a conditional R6K ori requiring the π protein for replication, expresses *sacB* for sucrose selection. | (1) |
| pRE-ΔsmpB | pRE112 derivative for *smpB* knock out in *A. veronii* C4, chloramphenicol resistance. | (1) |
| pTRG | Prey plasmid, 4.4 kb, ColE1 ori, *lpp/lac*-UV5 promoter, tetracycline resistance. | Stratagene |
| pTRG-smpB | pTRG derivative, expresses smpB with RNAP, tetracycline resistance | This study |
| pTRG-SN-peptides | pTRG derivative, expresses random peptide displaying on the *Staphylococcus aureus* nuclease (SN) with RNAP, tetracycline resistance. | (3) |
| pBT | p15A origin, chloramphenicol resistance, includes lac-UV5 promoter and λ cI ORF | Keysight Technologies,  CA, USA |
| pBT-SmpB | p15A origin, chloramphenicol resistance, expresses *smpB* with λ cI. | (1) |
| pBT (SmpB-G_11_S) | pBT-SmpB derivative, mutates G_11_S to AA. | (1) |
| pBT (SmpB-T_14_I) | pBT-SmpB derivative, mutates T_14_I to AA. | (1) |
| pBT (SmpB-F_26_I) | pBT-SmpB derivative, mutates F_26_I to AA. | (1) |
| pBT (SmpB-E_32_AG) | pBT-SmpB derivative, mutates E_32_AG to AAA. | (1) |
| pBT (SmpB-G_133_K) | pBT-SmpB derivative, mutates G_133_K to AA. | (1) |
| pBT (SmpB-D_138_KR) | pBT-SmpB derivative, mutates D_138_KR to AAA. | (1) |
| pBT (SmpB-K_152_) | pBT-SmpB derivative, mutates K_152_ to P. | (1) |
| pBBR-MCS-2 | Kanamycin resistance, broad-host-range cloning vector | (4) |
| pBBR-SmpB | Kanamycin resistance, contains full ORF region of *smpB* | This study |
| pBT-OmpA | p15A origin, chloramphenicol resistance,  expresses *ompA* with λ cI. | This study |
| pBT-ΔC-OmpA | p15A origin, chloramphenicol resistance,  expresses the truncation which is deficient in 114 residues of C-terminal OmpA with λ cI. | This study |
| pBT-ΔN-OmpA | p15A origin, chloramphenicol resistance,  expresses the truncation which is deficient in 193 residues of N-terminal ompA with λ cI. | This study |
| pBX-cmT | Modified pBT derivative, inserts to gene promoter for bacterial one-hybrid analysis. | (5) |
| pBX-cmT-*ompA* | pBT-cmT derivative, inserts to the promoter of *ompA* gene for bacterial one-hybrid. | This study |
| pEGFP-C2 | eGFP expression vector. | (6) |
| pDH113 | pBR322 origin, ampicillin resistance | This study |
| pDH116 | pDH113 derivative, pBR322 origin, ampicillin resistance, includes *ompA* promoter and the whole eGFP encoded region. | This study |
| pDH116-T_25_CT | The sites CAC mutated as TCT (+25 to +27 of the transcriptional start site in *ompA* promoter.) | This study |
| pDH116-T_52_GC | The sites CAT mutated as TGC (+52 to +54 of the transcriptional start site in *ompA* promoter.) | This study |
| pDH116-C-_17_AA | The sites TGG mutated as CAA (-17 to -15 of the translation initiation in *ompA* promoter.) | This study |
| pET-28a (+) | T7 promotor, kanamycin resistance, expresses His Tag . | Merck KGaA, Darmstadt, Germany |
| pET-SmpB | T7 promotor, kanamycin resistance, expresses *smpB* with His Tag. | (1) |
| pBT-SmpB | p15A origin, chloramphenicol resistance, expresses SmpB with λcI. | (1) |
| pTRG-SN | pTRG derivative, expresses SNase with RNAP, tetracycline resistance. | (1) |
| pTRG-SN-peptides | pTRG-SN derivative, expresses random peptide with RNAP, tetracycline resistance. | This study |
| pTRG-PA3 | pTRG derivative, expresses SN-PA3 with RNAP for interaction with OmpA, tetracycline resistance. | This study |
| pET-28a-SN | T7 promotor, kanamycin resistance, expresses *Staphylococcus aureus* nuclease (SNase) with His Tag. | This study |
| pET-28a-PA3 | T7 promotor, kanamycin resistance, expresses specific peptide aptamer SNase-PA3 with His Tag. | This study |

**Table S3 Primers in this study**

| **Primers** | **Sequnce(5’-3’)** | **Enzyme** |
| --- | --- | --- |
| pBBR-SmpB | 5’-G**GAATTC**ATGAGCAAAAAAAACAGTAAAAAC-3’  5’-CCC**AAGCTT**TTAGCCGCGATGCTTGTTCT-3’ | *Eco*R I  *Hind* III |
| pBT-OmpA | F:5’-CG**GAATTC**CATGGCTCAGGACAATACCTGGTA-3’  R:5’-GA**AGATCT**TTATTGGACAGGCTGGGAGAC-3’ | *Eco*R I  *Bgl* II |
| pBT-ΔC-OmpA | F:5’-CATG**CCATGG**GCGACAATACCTGGTACGCCG-3’  R:5’-CCG**CTCGAG**ACCTTGCTGACCGAAGCGA-3’ | *Nco* I  *Xho* I |
| pBT-ΔN-OmpA | F:5’-CATG**CCATGG**GCGATGTGCTGTTCGAGTTCAAC-3’  R:5’- CCG**CTCGAG**CAGATGGATTTCTACCCGGC-3’ | *Nco* I  *Xho* I |
| pBX-cmT-*ompA* | F:5’-GCTGCTGGTCATTTGAGGC-3’  R:5’-ATTTTCGCCTCTGCAATAAGTC-3’ |  |
| pDH116 | F:5’-GCCG**CGGCCG**GCTGCTGGTCATTTGAGGCT-3’  R:5’-CCTCGCCCTTGCTCACCATTACGGCAAGCATGGATTTATTCAT-3’  F:5’-ATGAATAAATCCATGCTTGCCGTAATGGTGAGCAAGGGCGAGG-3’  R:5’-GA**AGATCT**TTACTTGTACAGCTCGTCCATG-3’ | *Eag* I  *Bgl* II |
| pDH116-C_52_AT | F: 5’-AAGTGCGCACTCAGGCGCCTGCAACGACTTATTGCAGAGGCG-3’  R: 5’-CGCCTCTGCAATAAGTCGTTGCAGGCGCCTGAGTGCGCACTTT-3’ |  |
| pET-28a-PA3 | F:5’-CATG**CCATGG**GCGGTTACCCATACGACGTTCCAG-3’  R:5’-CCG**CTCGAG**GTCGATGTCAACTTGACCAG-3’ | *Nco* I  *Xho* I |

**Table S4 Primers for real-time PCR**

| **Gene** | **Primers for Real-time PCR** |
| --- | --- |
| GAPDH | F:5’-CAGTCGCGCTTGCAGATAGTCCT-3’ |
|  | R:5’-ACGCCTCCACCATCGATATCCTC-3’ |
| *ompA* | F:5’-CAGGACAATACCTGGTACGCC-3’  R:5’-CCAGCTCAAACGCCAGATTAG-3’ |

Supplementary References

[1] Liu, Z., Liu, P., Liu, S., Song, H., Tang, H., Hu, X. (2015). Small protein B upregulates sensor kinase *bygS* expression in *Aeromonas veronii*. *Front Microbiol*. 6, 579. doi: 10.3389/fmicb.2015.00579

[2] Dehio, C., Meyer, M. (1997). Maintenance of broad-host-range incompatibility group P and group Q plasmids and transposition of Tn5 in Bartonella henselae following conjugal plasmid transfer from *Escherichia coli*. *J Bacteriol*. 179, 538-540. doi: 10.1128/jb.179.2.538-540

[3] Liu, P., Chen, Y., Wang, D., Tang, Y., Tang, H., Song, H., Sun, Q., Zhang, Y., Liu, Z. (2016). Genetic Selection of Peptide Aptamers That Interact and Inhibit Both Small Protein B and Alternative Ribosome-Rescue Factor A of *Aeromonas veronii* C4. *Front Microbiol*. 7, 1228. [doi: 10.3389/fmicb.2016.01228](https://doi.org/10.3389/fmicb.2016.01228.)

[4] Kovach, M.E., Phillips, R.W., Elzer, P.H., Roop, R.M 2nd., Peterson, K.M. (1994). pBBR1MCS: a broad-host-range cloning vector. *Biotechniques*. 16, 800-802. doi: 10.1016/0378-1119(95)00584-1

[5] Guo, M., Feng, H., Zhang, J., Wang, W., Wang, Y., Li, Y., Gao, C., Chen, H., Feng, Y., He, Z.G. (2009). Dissecting transcription regulatory pathways through a new bacterial one-hybrid reporter system. *Genome Res*. 19, 1301-1308. doi: 10.1101/gr.086595.108.

[6] Qing, J., Xiao, H., Zhao, L., Qin, G., Hu, L., Chen, Z. (2014). Construction and characterization of an enhanced GFP-tagged TIM-1 fusion protein. *J Microbiol Biotechnol*. 24, 568-576. doi: 10.4014/jmb.1311.11077
